# On the Use of Tartar-Emetic Ointment in Chorea, &c.

**Published:** 1830-07

**Authors:** Charles Byrne

**Affiliations:** United States' Arsenal, near Baltimore.


### TARTAR-EMETIC OINTMENT.

*On the Use of Tartar-Emetic Ointment in Chorea, &c.* By Charles Byrne, m.d. United States' Arsenal, near Baltimore.*

Since the period when the illustrious Jenner drew attention to the external use of tartarized antimony, and produced so many interesting cases in proof of its efficacy in the treatment of a variety of troublesome diseases, very little experience has been offered to the profession as a test of the value of the remedy, or the soundness of his speculations. All that has been offered, however, is well calculated to increase our confidence in its virtues, and to entitle it to a more extended trial.

* American Journal of Med. Sciences.

In the eighth volume of the Medical Recorder, there is an account of a very extraordinary case of rotatio or chorea related by Mr. Hunter, of Glasgow, as successfully treated by rubbing the ointment of tartarized antimony into the scalp and spinal column. In the 33d number of the same Journal, a case of chorea is reported by Dr. Wharton, of Virginia, as cured by the same means.

The two cases of chorea which follow are the only ones which have occurred in my practice since the remedy has been suggested; and in both the result has been highly satisfactory.

August, 1828, I was called to visit Amanda Shaw, ætat. thirteen, sanguine temperament, small of her age, but not delicate in appearance; menstrual period not yet arrived. The arm and leg of the right side were the principal seat of muscular irregularity, although there was scarcely a muscle of the body that was not occasionly more or less disturbed; even the tongue refused at times to do its office, and very often performed it in a very imperfect manner. Many of the intellectual faculties were much impaired, particularly the memory; and the countenance betrayed a vacant, stupid expression. The appetite was bad, the tongue foul, and the bowels irregular, generally costive. Her mother stated that she had been in her present situation, with little variation, for the last two years; that during this time no regular medical assistance had been sought, but that she had herself tried a variety of remedies as recommended in "Buchan's Domestic Medicine," particularly blisters and purging; but with no perceptible advantage. As the case seemed to be a fair one for the trial of Dr. Hamilton's practice, I commenced the purgative plan, and continued it for two weeks, when the tongue became clean, and the bowels regular, but without any amelioration of the symptoms. I then gave tonics, decoction of valerian, and carbonate of iron; and finally touched the mouth with the blue pill, but was equally unsuccessful in all. I now prescribed the tartar-emetic ointment, to be applied to the whole spinal column, from the atlas to the sacrum. I ordered two drachms to be rubbed in three times a day, until a plentiful crop of pustules should be produced. On the evening of the second day the eruption began to appear, and from that time to the present, the patient has never been affected with the slightest irregularity of muscular motion. Her mental faculties in a short time resumed their wonted energy, and her health was perfectly restored. From the perseverance with which her mother continued to rub in the ointment after the appearance of the eruption, her back continued very sore for four or five weeks, longer perhaps than was necessary for the removal of the disease.

Case II. May 1829, I was called to E. Stansbury, a lad of twelve years old, good constitution, sanguine temperament, well grown. The symptoms in this case were in every respect similar to the first case. The patient had been labouring under the disease for nine months, during which time a great variety of remedies had been tried unsuccessfully by a medical gentleman of the neighbourhood. I immediately prescribed the ointment as in the other case, and with equal success, excepting that the right hand did not come entirely under the control of the will for twelve or fourteen days, but from that period there has been perfect immunity from the disease.

The following case of another description may perhaps be worthy of notice.

March 1st, 1829, I visited Sarah Leghorn, spinster, aged twenty-nine, labouring under acute pneumonia. This patient was tall, of delicate make, fair complexion, flat chest, and scrofulous family. From the preceding fall she had had a troublesome, hacking cough, pain in the breast, and occasional hæmoptysis. The most urgent symptoms were now speedily relieved by the usual remedies; but the cough, pain in the breast, and hæmoptysis, continued as before; a deep, well-defined, hectic flush appeared on the cheeks, and all the symptoms seemed to threaten a confirmed phthisis. I prescribed the Ung. Tart. Ant. to be rubbed in night and morning from the superior part of the sternum to its ensiform cartilage, (that being the seat of the pain and uneasiness,) until a plentiful crop of pustules should appear. I did not see the patient again for eight days, during which time she had persevered (having mistaken my directions,) to rub in the ointment until the whole of the space over which it was applied presented the appearance of one immense scab, open, and discharging matter all round its circumference. The pain and irritation produced by this immense sore had deprived the patient of sleep for the last four nights, and her sufferings were such that on my entering her room, (notwithstanding a very mild temper,) she reproached me in the bitterest manner, for having treated her so cruelly. By the application of fresh cream and emollient poultices, the burning sensation was relieved, and by the assistance of an anodyne she had some sleep on the succeeding night. The sore went on to discharge very profusely, and was so painful as to confine her to bed for four weeks. Masses of fungous flesh shot up all over its surface, which required the free use of caustic; and although no means were adopted to keep it open, it did not entirely heal until October, at which time it left the patient entirely free from all her threatening symptoms, and, from her own account, in much better health than she had been for years previously.

That an approaching consumption has been cured, or at least, suspended, in this case, by the use of this remedy, I have no room to doubt; but what *degree* of its application, short of the extreme to which it was accidentally carried, would have effected the same object, is a question of not so easy solution.

